# 甲氨蝶呤鞘内注射治疗脑膜癌病疗效和安全性的临床观察

**DOI:** 10.3779/j.issn.1009-3419.2016.05.08

**Published:** 2016-05-20

**Authors:** 熙 吴, 峻岭 李, 建平 肖, 渤彦 杨, 雷 于, 晓明 吴, 经海 万, 海燕 徐, 建新 孔

**Affiliations:** 1 100021 北京，中国医学科学院肿瘤医院综合科 General Department, Cancer Hospital Chinese Academy of Medical Sciences, Beijing 100021, China; 2 100021 北京，中国医学科学院肿瘤医院内科 Internal Department, Cancer Hospital Chinese Academy of Medical Sciences, Beijing 100021, China; 3 100021 北京，中国医学科学院肿瘤医院放疗科 Radiotherapy Department, Cancer Hospital Chinese Academy of Medical Sciences, Beijing 100021, China; 4 100021 北京，中国医学科学院肿瘤医院神经外科 Neurosurgery Department, Cancer Hospital Chinese Academy of Medical Sciences, Beijing 100021, China

**Keywords:** 脑膜癌病, 甲氨蝶呤, 鞘内注射, 安全性, Leptomeningeal metastases, Methotrexate, Intrathecal injections, Security

## Abstract

**背景与目的:**

脑膜癌病是中枢神经系统转移瘤的一种少见类型。近年来，随着恶性肿瘤治疗疗效的提高，患者生存期延长，脑膜癌病的发病率逐年增加，目前尚缺乏有效的治疗手段。本研究旨在探讨鞘内注射甲氨蝶呤（methotrexate, MTX）治疗脑膜转移的疗效、安全性和预后。

**方法:**

对27例脑膜转移患者的临床资料、脑脊液实验室检查进行回顾性分析，并分析鞘注化疗后的不良反应及预后。

**结果:**

27例脑膜转移患者接受鞘注化疗后，70.4%获得临床症状缓解，但脑脊液压力和脑脊液生化改变无统计学差异。55.6%患者无不适，25.9%出现下肢麻木、轻微疼痛，无严重不可逆的不良反应发生。本组患者中位生存期4个月。

**结论:**

鞘内注射MTX可改善脑膜癌病患者临床症状，无严重不良反应发生。

## Abstract (en)

**Background and objective:**

Leptomeningeal carcinomatosis is a rare type of metastatic tumors of the central nervous system. In recent years, with the improvement of neoplasms therapies and longer survival of patients by better systemic control, incidence of leptomeningeal metastases has increased every year. However, there is still lack of effective therapies. The aim of this study is to investigate the efficacy, security and prognosis of intrathecal chemotherapy with methotrexate (MTX) in the treatment of neoplastic meningitis.

**Methods:**

A total of 27 patients were enrolled. We investigated clinical features and cerebrospinal fluid (CSF) examination results retrospectively, and analyzed the adverse reactions as well as prognosis after intrathecal chemotherapy.

**Results:**

All 27 patients were treated by intrathecal MTX, 70.4% had clinical remission, however, there was no significant difference in CSF pressure and CSF biochemical changes. We observed that 55.6% patients were all appropriate, 25.9% appeared lower limb numbness and mild pain, no serious irreversible adverse reactions occurred. Median overall survival was 4 months.

**Conclusion:**

We suggest that intrathecal administration of MTX is associated with improvement of symptoms of leptomeningeal metastasis patients and no severe adverse events observed.

脑膜癌病是恶性肿瘤细胞弥漫性或多灶性浸润软脑膜和脑脊膜。大约3%-5%的实体瘤患者出现脑膜转移，常见于乳腺癌、肺癌、黑色素瘤，其中原发肺癌者占13%-29%^[1]^。脑膜癌病病情进展迅速，缺乏能显著改善生存率的治疗手段，预后差，未经治疗患者中位生存期4周-6周。常用治疗方法包括全脑放疗、鞘注化疗、全身化疗及脑室-腹腔分流手术等。我院2014年10月-2016年3月使用甲氨蝶呤（methotrexate, MTX）鞘内注射治疗脑膜癌病27例，所有患者均经脑脊液细胞学或头颅增强核磁确诊脑膜转移。现将这些病例的临床表现、实验室检查、疗效及不良反应进行回顾性分析，旨在提高脑膜癌病的治疗水平。

## 1 资料与方法

### 1.1 一般资料

纳入2014年10月-2016年3月在中国医学科学院肿瘤医院接受MTX鞘内注射治疗脑膜癌病27例，本组患者均有明确的肿瘤原发部位和病理类型。依据临床症状、头颅增强核磁和脑脊液细胞学检查确诊脑膜转移。男性12例，女性15例，年龄23岁-75岁，中位年龄50岁。肺癌25例，皮肤黑色素瘤1例，胃癌1例。小细胞肺癌4例，肺腺癌21例，胃腺癌1例。16例肺癌和1例黑色素瘤患者行基因检测，其中脑脊液基因检测5例，13例行肺癌原发灶或转移淋巴结检测，2例同时行脑脊液和肺原发灶基因检测，1例行血液基因检测。2例表皮生长因子受体（epidermal growth factor receptor, *EGFR*）20突变，11例*EGFR* 21突变，4例为EGFR野生型。6例以脑膜转移症状首诊。11例单纯脑膜转移，脑转移合并脑膜转移16例。20例患者合并其它部位转移，转移部位包括双肺、肝、骨、胸膜、脾、肾上腺、胸腹腔淋巴结、脊膜、脊髓等。确诊肿瘤至出现脑膜转移的时间为0个月-70个月，中位时间18个月。11例发生脑膜转移同时伴有全身肿瘤进展（表 1）。

**1:** 27例脑膜癌病患者的临床资料 The clinical data of 27 cases of patients with meningeal carcinomatosis

Characteristics	Data (*n*)	Percentage (%)
Gender		
Male	12	44.4 (12/27)
Female	15	55.6 (15/27)
Age (yr)		
Mean	50	
Range	23-75	
Primary tumor site		
Lung	25	92.6 (25/27)
Stomach	1	3.7 (1/27)
Skin	1	3.7 (1/27)
Pathohistology		
Adenocarcinoma	22	81.5 (22/27)
Small cell carcinoma	4	14.8 (4/27)
Melanoma	1	3.7 (1/27)
Gene mutation detection		
*EGFR* 20 mutation	2	11.8 (2/17)
*EGFR* 21 mutation	11	64.7 (11/17)
*EGFR* wild type	4	23.5 (4/17)
Metastatic site		
Meningeal	11	40.7 (11/27)
Meningeal and brain	16	59.3 (16/27)
Other organs	20	74.1 (20/27)
Time of onset to diagnosis of meningeal metastasis (mo)		
Mean	18	
Range	0-70	
Systemic disease state		
Stable disease	16	59.3 (16/27)
Progressive disease	11	40.7 (11/27)
ECOG score at admission		
ECOG≤2	11	40.7 (11/27)
ECOG>2	16	59.3 (16/27)
ECOG: Eastern Cooperative Oncology Group.		

### 1.2 临床表现

患者主要表现为脑、颅神经和脊神经根受损三组症状。其中头痛18例（66.7%），头晕15例（55.6%），恶心呕吐16例（59.3%），视神经受累（视力下降、复视、视物模糊）12例（44.4%），听神经受累（听力下降、耳鸣）7例（25.9%），面神经受累（面瘫）2例（7.4%），癫痫、抽搐4例（14.8%），行走不稳3例（11.1%），言语表达障碍2例（7.4%），记忆力减退2例（7.4%），2例（7.4%）有精神行为异常，表现为易怒、辱骂殴打家人。

### 1.3 脑脊液检查

27例患者接受鞘注化疗前均行腰椎穿刺检查，每次腰穿同时测定脑脊液压力、脑脊液常规和生化，每2-3次鞘注化疗后送检1次脑脊液细胞学。

### 1.4 治疗情况

1例因持续颅高压行腰大池引流术，3例行脑室腹腔分流术。10例诊断脑膜转移后接受全脑放疗。4例接受全身化疗，13例口服吉非替尼、厄洛替尼等靶向治疗，4例先后接受全身化疗与靶向治疗。所有27例均鞘内注射MTX（10 mg/次）和地塞米松（5 mg/次），每周1-2次。采用按摩式注射，缓慢推注且不断用脑脊液稀释，时间15 min以上。

### 1.5 观察指标

临床表现包括患者神经、精神方面症状。鞘注化疗前后脑脊液实验室检查。鞘注化疗后的不良反应。生存期（overall survival, OS）指诊断脑膜癌病到死亡或末次随访的时间。

### 1.6 统计学分析

应用SPSS 22.0软件对数据进行统计学处理，采用配对*t*检验，*P* < 0.05为差异有统计学意义。

## 2 结果

共12例接受3次-4次鞘内注射MTX，12例鞘注5次-9次，1例鞘注12次，1例鞘注15次，1例鞘注19次。

### 2.1 临床症状

19例（70.4%）临床症状缓解：1例眼球运动障碍改善，1例意识模糊好转，12例头痛明显减轻，5例视力、听力好转，2例精神障碍恢复。但8例（29.6%）症状无明显改善。

### 2.2 脑脊液实验室检查

27例患者中2例（7.4%）脑脊液细胞学阴性，其余25例（92.6%）脑脊液均找到癌细胞。3例脑脊液有小细胞癌细胞，1例找到黑色素瘤细胞，其余21例脑脊液找到腺癌细胞。鞘注化疗后部分患者脑脊液细胞学好转，7例转阴性，另有2例脑脊液中仅有几个退变的腺癌细胞（表 2）。治疗后脑脊液压力降低，脑脊液蛋白下降、葡萄糖回升，但均无统计学差异（*P*>0.05）（表 3）。

**2:** 脑膜癌病患者脑脊液细胞学变化 The changes of cerebrospinal fluid (CSF) cytology in patients with meningeal carcinomatosis

CSF cytological	Before IP	After IP
Without cancer cells	2 (7.4%)	9 (33.3%)
With cancer cells	25 (92.6%)	18 (66.7%)
Small cell carcinoma cell	3	0
Melanoma cell	1	1
Adenocarcinoma cell	21	17
IP: intrathecal chemotherapy.		

**3:** 鞘注化疗前后脑膜癌病患者脑脊液实验室检查变化 Laboratory examination of cerebrospinal fluid meningeal carcinomatosis patients before and after chemotherapy sheath injection

	CSF pressure (mmH_2_O)	CSF-Pro (mg/L)	CSF-Glu (mmol/L)
Before IP	194.26±97.19	735.65±629.80	2.56±1.23
After IP	173.70±107.11	712.35±662.71	2.95±1.49
*t*	1.139	0.303	1.566
*P*	0.265	0.764	0.130

### 2.3 鞘注MTX的不良反应

1例第3次鞘注后出现II度骨髓抑制（血小板59 g/L），1例第5次鞘注后Ⅲ度骨髓抑制（血小板35 g/L）。1例第2次鞘注后出现呼吸节律异常，对症治疗后好转。1例乏力明显。1例接受鞘注化疗过程中突然抽搐，终止治疗后迅速缓解，考虑与穿刺针仅部分进入蛛网膜下腔、MTX直接刺激脑脊膜相关。7例（25.9%）下肢麻木、轻微疼痛。15例（55.6%）无明显不适。

### 2.4 生存情况

2016年5月1日随访结束，随访时间2个月-23个月，1例失访，随访率96.3%。确诊脑膜转移后患者中位生存时间4个月，最短1个月，最长22个月。死亡10例（37.0%），4例死于呼吸衰竭，6例死于脑膜转移进展、脑疝。

## 3 讨论

脑膜癌病常见于恶性肿瘤晚期，病情进展迅速，预后差。好发于中老年，性别差异不明显。近年来脑膜癌病发病率逐渐升高，一方面是有效的抗肿瘤治疗使疾病自然病程延长，另一方面是某些新型抗肿瘤药很难透过血脑屏障，使中枢神经系统成为恶性细胞的庇护所。文献^[2]^报道脑膜转移发生的时间一般在原发肿瘤确诊后10个月-331个月，平均12个月。常为亚急性起病，50%患者的首发症状为脑部病变，临床表现有头痛、呕吐、后背痛；畏光、复视、面神经麻痹、听力下降、声音嘶哑、味觉改变；少数出现癫痫、行走不稳、精神障碍和记忆丧失。33%-75%同时合并脑（脊髓）实质转移。脑膜转移占原发癌症的比例分别是乳腺癌2%-5%，小细胞肺癌6%-25%，非小细胞肺癌1%-5%，黑色素瘤23%。肺癌脑转移的发生率约30%，5%-18%出现脑膜转移，约5%以脑膜癌病症状首次就诊^[3]^。本组研究中肺癌脑膜转移占92.6%，其中22.2%的患者以脑膜转移首诊，这与我国肺癌发病率居第一位有关。值得注意的是，40.7%患者发生脑膜转移时伴有全身肿瘤进展，59.3%的患者颅外稳定但已发生脑膜转移，可能与化疗药物透过血脑屏障浓度低相关。

研究^[1]^显示，未经治疗的患者总生存期约6周-8周，接受鞘注化疗的患者生存时间可延长至3个月-9个月。患者一般状况良好、肿瘤负荷小可从治疗中更多获益。此外，能接受脑放疗、全身化疗及鞘注化疗将有助于延长生存期^[4]^。Kumar等^[5]^推荐MTX鞘注化疗方案，诱导阶段：每周2次，每次鞘注10 mg-12 mg，共4周。巩固阶段：每周1次，每次鞘注MTX 10 mg-12 mg，共4周。后改为每2周1次，治疗8周。维持阶段：每4-8周1次，每次鞘注MTX 10 mg-12 mg。MTX在脑脊液中的半衰期约为4.5 h-8 h。如向脑室内直接注射MTX，建议减量50%，单纯脑室内注射MTX 6.25 mg/m^2^即可在蛛网膜下腔达到治疗浓度并维持48 h，且全身吸收极少^[6]^。鞘注化疗相对于单纯姑息治疗可延长脑膜癌病患者总生存时间。原发乳腺癌预后优于肺癌和黑色素瘤。目前尚无资料显示MTX联合阿糖胞苷或塞替派鞘注疗效优于MTX单药治疗。一项随机研究^[7]^显示，脑膜癌病患者每2周鞘注50 mg脂质体阿糖胞苷相较于每周鞘注2次10 mg MTX总生存期无统计学差异，但可显著延长神经系统病变进展时间（58 d *vs* 30 d）。本组每周1-2次鞘注MTX 10 mg/次，70.4%病例获得临床症状缓解，但脑脊液压力和脑脊液生化无显著变化。观察发现，治疗组中7例脑脊液细胞学转阴性，另有2例脑脊液中仅有几个退变的腺癌细胞，提示接受鞘注化疗后脑脊液细胞学好转。但需要注意的是，鞘注化疗后1次检查脑脊液细胞学阴性并不意味治疗有效。Chamberlain等^[8]^提出脑脊液细胞学完全缓解的定义是：间隔1周以上、连续2次细胞学阴性，且持续时间超过1个月。部分缓解定义为：上述相同条件下脑脊液细胞学从阳性转为可疑阳性。有报道显示，脑脊液细胞学变化与患者总生存期不相关，且鞘注化疗后脑脊液细胞学转阴性者可能短时间内再次转为阳性。本研究中1例脑脊液细胞学转阴性后12个月脑脊液中再次找到癌细胞，另1例6个月后脑脊液细胞学再次阳性。

鞘注化疗的主要并发症有颅内感染和脑脊液循环梗阻。鞘注MTX引起的骨髓抑制可使用四氢叶酸钙解救。罕见发生化学性脑膜炎，治疗相关的神经毒性亦较少出现，但可导致亚急性脑白质变性^[5]^。颅高压和脑积水是影响鞘注化疗疗效的原因之一，放射性核素脑池造影检查发现，40%-60%的脑膜癌病患者存在脑脊液循环障碍。脑脊液循环异常阻碍了药物在脑室内的均匀分布，导致跨室管膜药物浓度梯度增高，增加了脑白质变性病发生的概率^[9]^。本研究中55.6%的患者接受鞘注化疗后无明显不适，25.9%出现下肢麻木、轻微疼痛。1例治疗过程中抽搐，考虑与操作不当相关。另有1例一过性呼吸节律异常、对症治疗后好转，尚有2例发生骨髓抑制。治疗组患者中未观察到有急性肺水肿、横贯性脊髓炎等严重并发症的发生。

脑膜癌病患者预后较差，本组患者中位生存期4个月，最长22个月，采用全身化疗、靶向治疗、中枢神经系统放疗、鞘注化疗等联合治疗手段有助于延长生存期。

综上所述，脑膜癌病是肿瘤晚期的严重并发症之一，鞘内注射MTX可显著改善患者临床症状，但脑脊液压力、脑脊液葡萄糖和蛋白质无明显变化。鞘注化疗是治疗脑膜癌病的安全有效方法之一。
